# Quantitative Analysis of Carbonic Anhydrase IX Uncovers Hypoxia-Related Functional Differences in Classical Hodgkin Lymphoma Subtypes

**DOI:** 10.3390/ijms20143463

**Published:** 2019-07-15

**Authors:** Orsolya Matolay, Lívia Beke, Andrea Gyurkovics, Mónika Francz, Gabriella Varjasi, László Rejtő, Árpád Illés, Judit Bedekovics, Gábor Méhes

**Affiliations:** 1 Department of Pathology, Faculty of Medicine, University of Debrecen, H-4032 Debrecen, Hungary; 2 Department of Pathology, Jósa András Teaching County Hospital, H-4400 Nyíregyháza, Hungary; 3 Hematology Unit, Jósa András Teaching County Hospital, H-4400 Nyíregyháza, Hungary; 4 Department of Hematology, Faculty of Medicine, University of Debrecen, H-4032 Debrecen, Hungary

**Keywords:** classical Hodgkin’s lymphoma, digital image analysis, virtual slide, hypoxia, necrosis, CAIX, CD30

## Abstract

Upregulation of carbonic anhydrase IX (CAIX) was found to be associated with unfavorable prognosis and resistance to treatment in a broad spectrum of malignancies, recently also in classical Hodgkin’s lymphoma (cHL). As demonstrated, variable CAIX expression in a significant number of cHL cases was associated with poor treatment response. The current study focused on the quantification CAIX immunopositivity and its relative expression compared to the total CD30+ neoplastic pool using digital image analysis. One hundred and one lymph node samples featuring cHL histology were analyzed for both CD30 and CAIX by immunohistochemistry. Whole histological slides were scanned and immunopositivity was determined as the histoscore (H-score) using the DensitoQuant software module (3DHistech Kft., Budapest, Hungary). CAIX positivity was observed in the HRS-cells of 56/101 cases (55.44%) and frequently observed in the proximity of necrotic foci. CAIX H-scores were highly variable (range: 2.16–90.36, mean 18.7 ± 18.8). Individual CAIX values were independent of the much higher CD30 values (range 3.46–151.3, mean 52.37 ± 30.74). The CAIX/CD30 index proved to be the highest in the aggressive lymphocyte-depleted (LD) subtype (CAIX/CD30: 0.876). The CAIX expression and the CAIX/CD30 relative index can be precisely determined by image analysis, and values reflect the extent of a tumor mass undergoing hypoxic-stress-related adaptation in the most aggressive forms of cHL.

## 1. Introduction

Classical Hodgkin’s lymphoma (cHL) is a unique B-cell derived lymphatic malignancy featured by characteristic morphology. As a key feature, cHL presents with highly enlarged atypical neoplastic cells (Hodgkin–Sternberg–Reed cells, HRS cells) surrounded by a rich reactive cell infiltrate, consisted of lymphocytes, plasma cells, histiocytes, and stromal elements. Moreover, the infiltrate may be accompanied by necrotic foci. According to the WHO 2016 classification, cHL can be further subdivided into four histological subgroups: Nodular sclerosis (NS), mixed cellularity (MC), lymphocyte rich (LR), and lymphocyte depleted (LD) cHL [1,2]. NS is the most frequent subtype of cHL accounting for approximately 80% of the cases and characterized by fibrosis and the nodular arrangement of the neoplastic HRS-cell component. MC is the second most frequent type composed of a mixture of neoplastic cell clusters and a high variety of activated inflammatory cells. In contrast, the LR subtype is dominated by monotonous fields of small lymphocytes and only limited numbers of HRS cells. LD histology is associated with the worst clinical prognosis. The paucity of the lymphoid component and the abundance of the HRS cells in a solid tumor-like fashion is typically observed here. HRS-cells show an aberrant immunophenotype highlighted by massive CD30 expression in virtually all cases and further, the loss of leukocyte common antigen (CD45RA) and the B-cell marker CD20 [3]. Overexpression of CD30 in the HRS-cells is highly characteristic in cHL and is one of the key diagnostic features [4,5]. CD30, a 120 kD membrane-associated glycoprotein is a member of the tumor necrosis receptor family and has a crucial role in antiapoptotic signaling and in cytokine expression [5,6,7].

Hypoxia may frequently develop in progressive solid tumors both in a transient or a long term fashion. Hypoxia-inducible transcriptional Factor 1α (HIF1α) is a major regulator of the functional and metabolic adaptation through the activation of multiple signaling pathways [8,9]. Carbonic anhydrases and especially carbonic anhydrase IX and XII (CAIX and CAXII) have a pivotal role in the adaptation to hypoxia-driven acidosis, therefore, they are also considered as endogenous hypoxia markers. The effective enzymatic neutralization of the intracellular acidosis due to increased lactate production consequently contributes to the decrease of the extracellular pH [10]. CAIX upregulation was found to be associated with cellular quiescence and extended survival and was reported to be related with unfavorable outcome and disease progression in e.g., breast and stomach cancer [11,12,13,14].

In our previous study, we examined the expression of CAIX in HRS-cells of cHL samples using immunohistochemistry and concluded that high CAIX expression influences disease outcome and may play a special role in the short term failure of the applied chemotherapy [15]. However, due to the variable number and uneven distribution of neoplastic HRS cells in the background of the non-neoplastic inflammatory cells, the exact quantification of CAIX immunopositivity was not enabled by conventional light microscopy. Therefore, we decided to perform a quantitative evaluation of HRS cells using digitalized slides enabling objective selection and segmentation of specific immunolabeling in the relevant tissue areas. Whole slide digital analysis (WSD) provides the visual control of the neoplastic cells and their microenvironment for the same time, which is especially challenging in cHL [16,17]. Literature is available on image analysis approaches in a wide range of tumors, however, the number of studies on cHL is still limited [18,19,20]. According to our current approach, CD30 IHC labeling was applied as the most accurate way to represent the total amount of HRS cells which can be used as a reference for further functional studies. In the present work, the expression of CD30 and CAIX was measured in parallel within digital slides to establish the relation between the malignant cell burden (represented by CD30 positive HRS cells) and adaptive changes highlighted by CAIX-related immunolabeling.

## 2. Results

### 2.1. CD30 and CAIX Expression in Hodgkin’s Lymphoma

**Figure 1:**
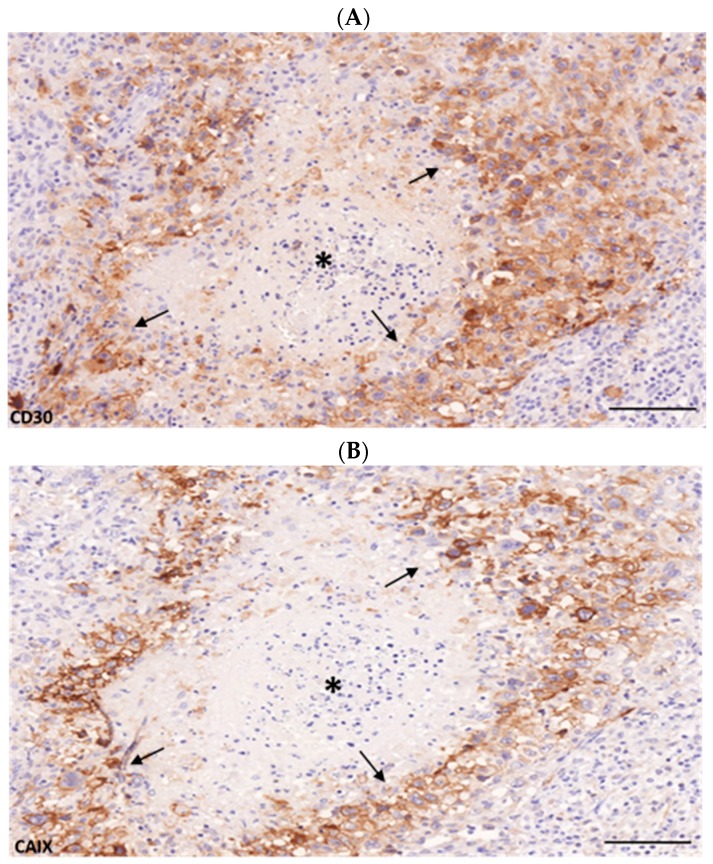
CD30 expression (**A**) representing Hodgkin–Sternberg–Reed (HRS) cells and partially carbonic anhydrase IX (CAIX) positivity (**B**) demonstrating hypoxia-related adaptation in a subset of the neoplastic compartment surrounded by reactive cell masses characteristic for classical Hodgkin’s lymphoma (cHL). Variable levels of membrane/cytoplasmic CAIX expression could be observed in the neoplastic HRS cells while the non-neoplastic background remained virtually negative. Samples with necrosis showed intense CAIX expression in the perinecrotic area (arrows). Asterix indicates the center of the necrotic area. Scale bar = 100 μm.

cHL samples included all showed a robust and clear CD30 immunostaining which selectively labeled the full spectrum of HRS cells as expected (Figure 1A). CAIX immunohistochemistry provided a specific but highly variable reaction on a subset of large atypical cells with HRS morphology. Virtually no other cell types were found to be positive (Figure 1B). The dominantly strong membrane and weak cytoplasmic immunostaining allowed a simple differentiation between positive and negative HRS cells in the microscope with only little limitation. Out of the 101 samples analyzed, 56 cases (55.44%) were considered as positive for CAIX, the residual 45 cases (44.5%) showed no clear sign of CAIX-related immunolabeling. Within the CAIX-positive samples, areas with labeled and unlabeled HRS cells were also seen, sometimes with clear cluster formation. Moreover, significant intercellular heterogeneity was also observed. As expected, in cases with morphologically manifest tumor necrosis, the perinecrotic areas were typically enriched. Necrotic foci were usually classically surrounded by sheaths of CAIX+ neoplastic cells representing the adaptation zone (Figure 1B).

While necrosis was found only in 26.7% (27/101) of the evaluated samples, 85.0% of these samples presented with CAIX-positive neoplastic cells (Table 1).

The total study cohort of 101 samples covered the histological spectrum of cHL and consisted of 70 NS, 20 MC, 7 LR, and 4 LD cases presenting with different CAIX expression rates (Table 1). Of note, cases with LD and NS subtypes were highly positive for CAIX with rates of 4/4 (100%) and 46/70 (65.71%), respectively. In contrast, MC and LR subtypes were only rarely positive (4/20, 20.0% and 2/7, 28.57%).

### 2.2. Measurement of CAIX and CD30 Expression on Digital Slides

**Figure 2:**
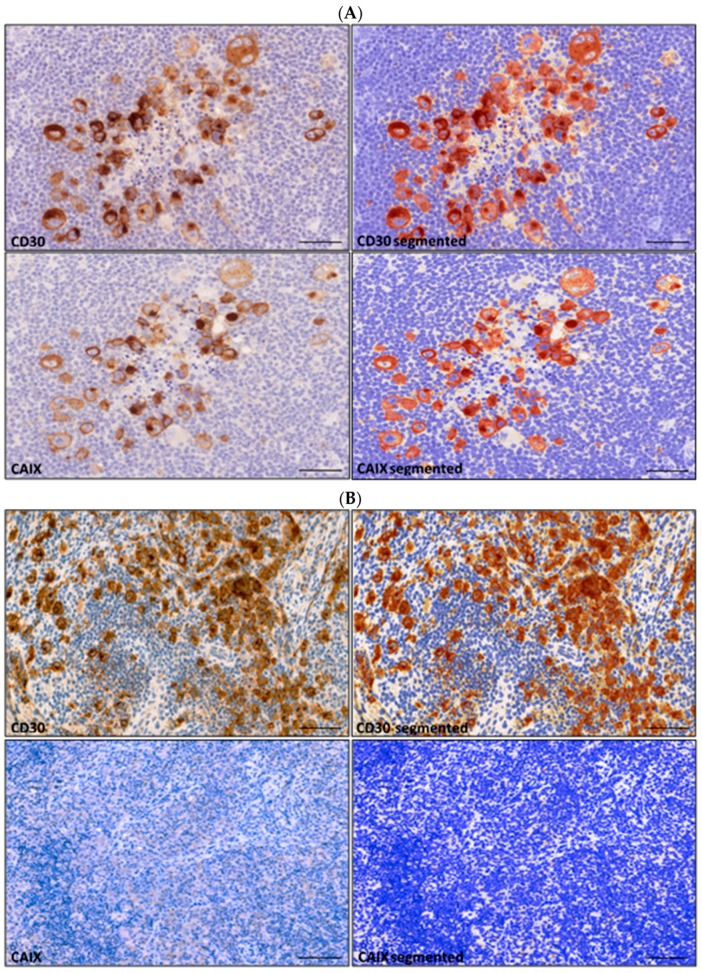
DensitoQuant digital image analysis of CD30 and CAIX-immunolabeling in CAIX positive (**A**) and negative (**B**) classical Hodgkin’s lymphoma. Immunopositivity of large atypical HRS cells (left) is highlighted as pseudocolored pixels (yellow/orange/red) following digital segmentation (right). Number and intensity of positive pixels were expressed as individual H-score for each sample. Scale bar = 100 µm.

Staining intensity measurements were done following the positive selection for both CD30 and CAIX in all suitable areas using the DensitoQuant module of the QuantCenter 2.0 (Figure 2). H-scores were determined by measuring the total pixel counts for each of the three intensity levels (high, medium, and weak staining intensities). A cut-off level of immunopositivity (H-score> 1.0) was set following the measurement and careful evaluation of immunohistochemistry (IHC)-negative non-cHL lymph node control samples treated and stained the same way. The utility of this cut-off value proved to be valid as it was never reached in CAIX-negative cHL slides.

Image analysis measurement of CD30 resulted in highly variable values (H-scores between 3.38 and 151.33, mean: 45.7 ± 29.3). CAIX H-score values were generally very low (between 0.01 and 0.99, mean 0.37 ± 0.29) in the negative samples (*n* = 45) and proved to be significantly higher in the positive samples (H-score range: 2.16–90.36, mean 18.7 ± 18.8) (*n* = 56) determined by conventional light microscopy.

**Figure 3:**
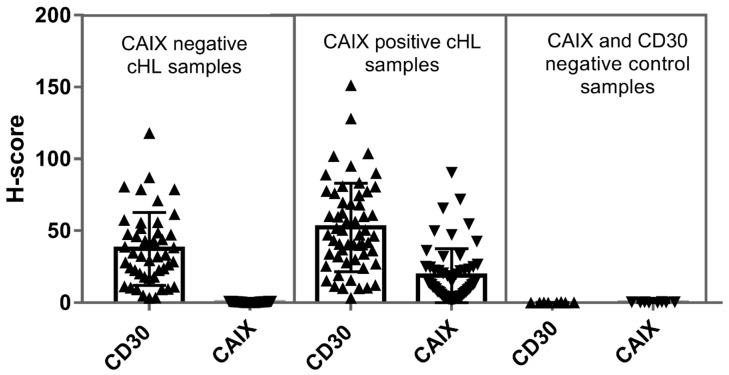
Distribution and mean values of CD30 and CAIX-related H-scores determined by image analysis. The figure demonstrates the individual H-score values determined for the CAIX-negative (*n* = 45) and CAIX-positive (*n* = 56) groups defined by light microscopy. As negative control CD30- and CAIX-double negative nonclonal lymphoproliferations were measured (*n* = 8). The CD30 H-scores representing the total HRS cell burden proved to be higher in CAIX-positive compared to the CAIX-negative samples according to the Mann–Whitney test (*p*-value = 0.008, statistically significant).

To explore the relation between the total neoplastic CD30+ cell mass and the occurrence of CAIX+ cell clusters we first compared the total amount of CD30-staining between the CAIX-negative and the CAIX-positive slides. Samples lacking CAIX-positivity presented with a significantly lower CD30-score (H-score range: 3.38–118.2, mean 37.38 ± 25.34) compared to CAIX-positives (CD30 H-score range: 3.46–151.33, mean 52.37 ± 30.74). Accordingly, the CD30+ mass proved to be significantly increased in association with the occurrence of CAIX+ fraction (Mann–Whitney U-test, *p* = 0.008) (Figure 3, Table 2).

### 2.3. Relation of CAIX to CD30 in cHL

CAIX immunopositivity proved to be highly variable, but CAIX H-score values (range: 2.16–90.4, mean 18.7 ± 18.8) appeared generally lower than CD30 H-scores (range: 3.46–151.33, mean 52.37 ± 30.74) suggesting the partial upregulation of the enzyme according to the functional diversity of HRS cell subpopulation (Figure 3).

**Figure 4:**
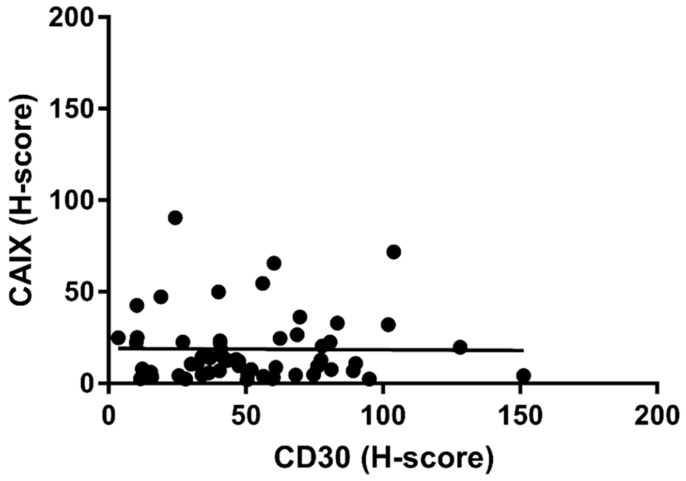
The CD30 related H-score representing the HRS cell burden did not correlate with the amount of CAIX expression in individual CAIX+ cHL samples (*p*-value = 0.4755, Spearman rs: −0.008).

While the CD30+ burden was generally increased in the CAIX+ compared to the CAIX− samples, there was no further positive correlation between the amount of CD30+ mass and the extent of CAIX+ cells within individual CAIX+ cases (*p*-value = 0.475, Spearman rs: −0.008) (Figure 4).

The relation between individual CAIX and CD30 H-score values could be also established on a case-by-case basis. CAIX H-scores exceeding CD30 values were found in altogether 6/56 (10.7%) samples and these were generally associated either with low HRS-counts or with unexpected low CD30-staining intensities.

Independent of CAIX expression, the overall HRS cell burden reflected by the CD30 H-score proved to be the highest in the LD subtype (*n* = 4, range: 24.29–109.95, mean: 63.1 ± 32.9). A wide range of CD30 positivity was observed in the NS (*n* = 70, range: 2.16–128.3, mean: 46.85 ± 27.5) and in the MC subtypes (*n* = 20, range: 3.38–151.3, mean: 45.65 ± 35.2) showing similar distribution. The lowest CD30 H-scores were detected in the LR subtype as expected (*n* = 7, range: 2.518–28.5, mean: 17.5 ± 8.8).

**Figure 5:**
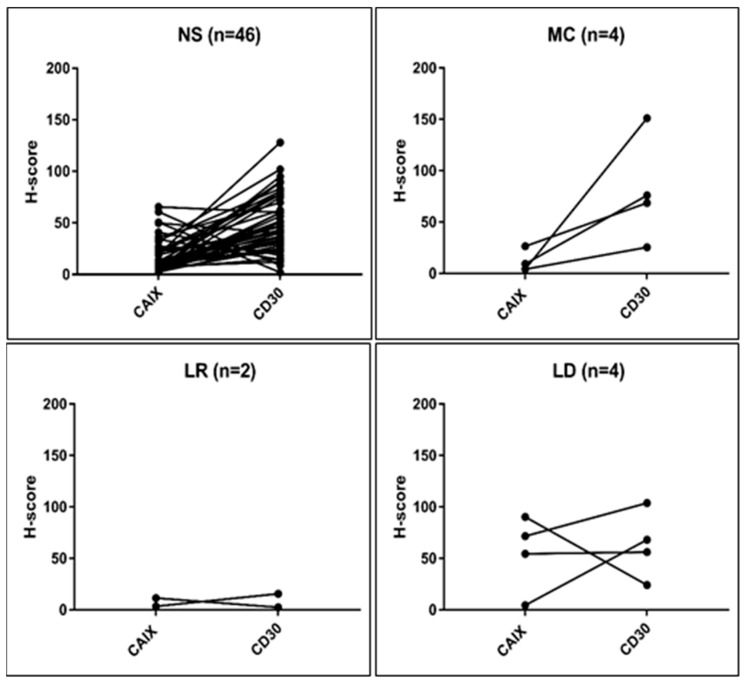
CAIX and CD30 H-scores measured in serial sections of CAIX-positive cHL (*n* = 56) according to the histological subtypes. CAIX H-score exceeded CD30 in six samples which was associated with low HRS-counts or low CD30 staining intensity.

Figure 5 graphically presents the specific relation of the matched values according to the histological cHL subgroups. The highest CAIX-levels (mean 55.3 with a maximum value of 90.36) were demonstrated in the LD while the lowest (mean 7.64, maximum 9.2) in the LR subtype with significant variability, as observed in all other histological groups.

To better characterize the intratumor variability, the relative positivity rate, expressed by the CAIX/CD30 ratio was also calculated for all cHL subtypes. The mean ratio was 0.23 ± 0.57 for all evaluated samples and 0.35 ± 0.61 for CAIX-positive samples. Significant differences among the histological variants could be demonstrated (Table 2). The LD subtype showed the highest relative immunopositivity for CAIX as the CAIX/CD30 index reached 0.87. While in NS cases the CAIX-relative index was 0.35, the MC subtype presented with modest relative positivity (0.13). Although the relative CAIX/CD30 index remained high (0.83) in the LR subtype, the low number of cases and rare positive events within the samples measured did not allow further statistical considerations.

## 3. Discussion

Hypoxia-related adaptation and focal necrosis are both important hallmarks of malignant progression [8,21,22,23]. Although ischemia and tumor necrosis in histological conditions is considered as a progressive sign associated with massive proliferation activity in selected cancer types (e.g., glioblastoma), adaptive mechanisms to hypoxia are rarely considered and the clinical utility of hypoxia-related signs, such as CAIX expression, is still underrated. Unlike the most common malignancies, there is only a limited number of studies about hypoxia-associated changes in lymphatic neoplasias, including cHL [15]. cHL belongs to the relatively mild forms of lymphomas with overall survival rates of above 80% [1,2]. The remaining cases, on the other hand, may show rapid progression, relapse and aggressive transformation with high cell proliferation rates and frequent tissue necrosis. While the clinical impact of necrosis could not be established so far, in a recent work we presented a statistically significant correlation between CAIX expression determined by immunohistochemistry and the unfavorable response to first-line therapy in cHL. We also stated that the exact quantification of the CAIX+ compartment is challenging as CAIX appears regionally in a highly variable and heterogeneous fashion.

In the present study, we aimed to determine the expression levels of CAIX related to the CD30-expressing neoplastic cell mass present in the sample. CD30 immunostaining is one of the basic markers which presents the full spectrum of transformed HRS cells characteristic for cHL in histological sections. Despite significant intratumor variability, the CD30 staining proved to be a robust and useful approach to identify and quantify the malignant cell compartment. In order to standardize the immunoscoring, we used the DensitoQuant digital analysis tool (3DHistech, Budapest, Hungary) following digital slide scanning. Pixel-based measurements enabled the exact quantification of CD30-related immunopositivity which could be expressed as H-scores for each sample. Virtually CD30-negative lymph node sections enabled to determine the background reaction which was negligible in these measurements. According to this setting, the CD30 values, measured by digital image analysis represented the neoplastic cell burden within the individual sections. This approach is in line with the limited amount of image analysis approaches which examined Hodgkin’s lymphomas on a slide-based digital platform [19,20,24]. As next, CAIX-related immunopositivity could be determined and individual H-scores were generated using an almost identical setup. During these measurements, CAIX was not detected in lymphatic changes other than cHL and results regarding positive staining were in accurate agreement with those of the conventional microscopy.

The presented method enabled the precise measurement and comparison of the IHC reaction for both CD30 and CAIX obtained in clinical samples. According to our data, CD30-related H-scores were generally higher than the CAIX H-scores with only a few exceptions. This was explained by unusual weak CD30 staining intensities of unknown nature. As a major limitation of any of the IHC methods, we had to consider the biological variability of patient samples provided for analysis.

Most importantly, more than half of the evaluated samples (56/101, 55.44%) presented with CAIX-expression, which seemed to be strictly related to the HRS cells. CAIX-positivity separated a subfraction of the total CD30+ HRS cell mass strongly suggesting that CAIX expression was not an integral feature of the transformed cells. Moreover, the CAIX staining was frequently associated with necrotic tissue foci further supporting the functional role of the enzyme in adaptation to hypoxia.

Interestingly, we found a significant difference (Mann–Whitney analysis, *p* < 0.0001) in the CD30+ tumor-cell burden between the CAIX+ and CAIX− cHL group (Figure 3). This allows the interpretation that CAIX upregulation occurs in the progressive, tumorous forms of cHL and that it is associated with the survival advantage of HRS-cells. However, in a further evaluation CAIX-scores did not follow the increase of CD30 (CAIX+ samples) and no relevant positive correlation was proven (Figure 4).

CAIX-expression could also be determined in a relative fashion for the first time as matched H-scores for the CD30-positive malignant cell mass were provided. In general, only a minor fraction of the HRS-cells presented with CAIX-reaction (mean: 0.35) but this could be further differentiated according to the histological lymphoma subtype. The most unfavorable clinical prognostic group (LD) provided the highest relative CAIX-expression (mean 0.87) as expected. The most frequent intermediate group (NS) presented a significantly lower value (mean 0.35), and the mildest form (MC) of the disease showed moderate relative H-score ratios (mean 0.13) (Table 2). The lymphocyte-rich subtype could not be exactly considered due to the low case number (n = 2) and the very low frequency of the HRS cells (H-score < 10) providing unsatisfactory data for statistical evaluation.

The general clinical interest for biological variables and functional differences within the otherwise ordinary-appearing malignant proliferations is increasing. The current study focused on the possibilities and challenges of the digital analysis to determine adaptive changes of an unselected group of malignant lymphoproliferation. Although our approach in the current phase is exploratory, we could measure major differences in routine pathological samples in relation to histological features and the tumor cell mass. This used to be a particularly challenging issue in Hodgkin’s lymphoma as CD30+ malignant B-cells (termed as HRS cells) are provided in a scattered fashion embedded in a rich reactive/inflammatory lymphatic environment. As stated, CAIX overexpression was a feature of the malignant cell compartment while the accompanying inflammatory and stromal elements remained virtually negative. Although there was a close relation of CAIX-expression with hypoxic necrosis in the aggressive histological variants, obvious signs of necrosis were present only about half of the CAIX+ cases (27/56, 48.21%). CAIX in cHL, therefore, most probably occurs in a dynamic fashion as an adaptive feature induced by hypoxic stress. The upregulation of the enzyme suggests the activation of protective mechanisms in metabolically challenged atypical HRS cells. This might be sorely essential as this enlarged neoplastic cell type demands basically different O_2_ and energy supply exceeding that of the non-neoplastic microenvironment.

In summary, the results show that the CAIX-positive cells compose a subfraction of the CD30-positive cells, and indicate a significant functional heterogeneity within the malignant clone. As CAIX-related cancer cell adaptation potentially contributes to an aggressive and resistant phenotype, analyses of this type open the way for alternative therapeutic considerations.

## 4. Materials and Methods

### 4.1. Patients and Specimens

We identified 101 lymph node samples diagnosed with cHL between 1999 and 2018 which were suitable for the analysis. Only samples from the time of the initial diagnosis were used. Core needle biopsies were avoided in this study. Histological diagnosis of cHL was given according to 2016 WHO classification.

The archived tissue samples and basic clinical data derived from the pathology departments of the University of Debrecen in Debrecen, Hungary, and the Jósa András Teaching Hospital, Nyíregyháza, Hungary. Ethical approval for this study was obtained from the Local Regional Ethics Board (60355-2016/EKU).

### 4.2. Immunohistochemistry

Representative lymph node biopsy samples were selected to include typical neoplastic cells. Immunohistochemistry was done as usual on serial sections using the antibody clone Ber-H2 (Dako-Agilent, Glostrup, Danemark) for CD30 and the rabbit polyclonal antibody clone NB100-417 (Novus Biologicals, Littleton, CO; final dilution 1:2000) for CAIX. Visualization was done by the diaminobenzidene)(DAB)chromogen using the Bond Max automated system (Leica, Germany). The result of the IHC reaction was carefully evaluated by light microscopy. To avoid potential false positivity, samples with critically low numbers of positive events (less than 10 nucleated immunopositive cells) were excluded.

### 4.3. Digital Image Analysis and IHC Scoring

The IHC stained slides were digitalized with the Pannoramic MIDI Scanner (3DHistech Ltd., Budapest, Hungary) and the scanned virtual slides were further analyzed. The area of the measurement was defined for each lymph node section using the CaseViewer slide management software (3DHistech Ltd., Budapest, Hungary). The size and shape of the annotation was highly individual depending on the characteristics of the biopsy sample. Irrelevant areas, such as artifacts, glue and stain residues, air bubbles, tissue folds, etc., were excluded digitally.

As next, the QuantCenter Software 2.0 (3DHistech Ltd., Budapest, Hungary) was applied in order to identify and score CD30 and CAIX positive cells. This allowed determining individual IHC values and the CAIX/CD30 relative index representing the whole sample. The DenstioQuant algorithm of QuantCenter is a stain-intensity measurement module enabling a user-controlled whole slide analysis on a pixel basis. As an initial step, the IHC signal was controlled and manually optimized to get reliable monochrome intensity values. Next, pixel intensity levels of the positive reaction were scaled and displayed in three levels: Weak (yellow), moderate (orange), and strong (red). The background of negative cells was represented by the blue-stained nuclei and unstained pixels in white color. The module automatically calculated histoscores (H-score) using individual pixel intensity levels and the total area of pixels. Ratios for negative, weak, moderate, and strong positives, as well as background pixels were generated. The H-score representing specific immunolabeling for each slide was used for further evaluation.

### 4.4. Statistical Analysis

Associations between paired samples were calculated by nonparametric Wilcoxon paired *t*-test and for group-based comparisons, the Mann–Whitney U-test was applied. Nonparametric Kruskal–Wallis analysis and Spearman’s nonparametric correlation was performed to calculate differences between CAIX and CD30 values in the matched sample sets using the Graph Pad Prism 7 (GraphPad Software, La Jolla, CA, USA). Statistical significance was set at *p*-values was less than 0.05.

## Figures and Tables

**Table 1:** Characteristics of the 101 cHL cases evaluated for CAIX and CD30 immunopositivity by both light microscopy and digital image analysis (H-score). N: number, CAIX: Carbonic anhydrase, cHL: classical Hodgkin’s lymphoma, NS: Nodular sclerosis, MC: Mixed cellularity, LR: Lymphocyte rich, LD: Lymphocyte depleted.

	N	%
**CAIX positivity**		
Total	56/101	55.44
NS	46/70	65.7
MC	4/20	20.0
LR	2/7	28.6
LD	4/4	100.0
**Necrosis**		
CAIX+	23/27	85.0
CAIX-	4/27	14.8
**Female**		
CAIX+	30/48	62.5
CAIX-	18/48	37.5
**Male**		
CAIX+	26/53	49.0
CAIX-	27/53	50.9
B-symptoms		
CAIX+	29/48	60.4
CAIX-	19/48	39.5
Age		
Mean	36.66	
<60 years	87/101	86.1
>60 years	14/101	13.8

**Table 2:** H-score based relative CAIX-expression (CAIX/CD30) according to the different histological subtypes of cHL (* only two samples, low cell numbers).

	n	CD30 Mean	CAIXMean	CAIX/CD30 Ratio
All cases	101/101	45.7 ± 29.3	10.54 ± 16.65	0.23 ± 0.57
CAIX- total	45/101	37.38 ± 25.34	0.37 ± 0.29	0.01 ± 0.01
CAIX+ total	56/101	52.37 ± 30.74	18.70 ± 18.88	0.35 ± 0.61
NS	46/70	49.86 ± 27.49	17.46 ± 15.8	0.35 ± 0.57
MC	4/20	80.46 ± 52.23	11.13 ± 10.62	0.13 ± 0.20
LR *	2/7	9.114 ± 9.325	7.64 ± 5.63	0.83 ± 0.60
LD	4/4	63.16 ± 32.9	55.3 ± 36.8	0.87 ± 1.12

## Abbreviations

CAIX	Carbonic anhydrase IX
cHL	Classical Hodgkin’s lymphoma
HIF1α	Hypoxia Inducible transcriptional Factor-1α
HL	Hodgkin lymphoma
HRS	Hodgkin–Sternberg–Reed cells
IHC	Immunohistochemistry
LD	Lymphocyte depleted
LR	Lymphocyte rich
MC	Mixed cellularity
NS	Nodular sclerosis
WSD	Whole slide digital analysis
